# Insights into the molecular mechanisms of Huangqi decoction on liver fibrosis via computational systems pharmacology approaches

**DOI:** 10.1186/s13020-021-00473-8

**Published:** 2021-07-23

**Authors:** Biting Wang, Zengrui Wu, Weihua Li, Guixia Liu, Yun Tang

**Affiliations:** Laboratory of Molecular Modeling and Design, School of Pharmacy, East China University of Science and Technology, Shanghai, 200237 China

**Keywords:** Huangqi decoction, Liver fibrosis, Mechanism of action, Metabolomics, Molecular docking, Network pharmacology

## Abstract

**Background:**

The traditional Chinese medicine Huangqi decoction (HQD) consists of *Radix Astragali* and *Radix Glycyrrhizae* in a ratio of 6: 1, which has been used for the treatment of liver fibrosis. In this study, we tried to elucidate its action of mechanism (MoA) via a combination of metabolomics data, network pharmacology and molecular docking methods.

**Methods:**

Firstly, we collected prototype components and metabolic products after administration of HQD from a publication. With known and predicted targets, compound-target interactions were obtained. Then, the global compound-liver fibrosis target bipartite network and the HQD-liver fibrosis protein–protein interaction network were constructed, separately. KEGG pathway analysis was applied to further understand the mechanisms related to the target proteins of HQD. Additionally, molecular docking simulation was performed to determine the binding efficiency of compounds with targets. Finally, considering the concentrations of prototype compounds and metabolites of HQD, the critical compound-liver fibrosis target bipartite network was constructed.

**Results:**

68 compounds including 17 prototype components and 51 metabolic products were collected. 540 compound-target interactions were obtained between the 68 compounds and 95 targets. Combining network analysis, molecular docking and concentration of compounds, our final results demonstrated that eight compounds (three prototype compounds and five metabolites) and eight targets (CDK1, MMP9, PPARD, PPARG, PTGS2, SERPINE1, TP53, and HIF1A) might contribute to the effects of HQD on liver fibrosis. These interactions would maintain the balance of ECM, reduce liver damage, inhibit hepatocyte apoptosis, and alleviate liver inflammation through five signaling pathways including p53, PPAR, HIF-1, IL-17, and TNF signaling pathway.

**Conclusions:**

This study provides a new way to understand the MoA of HQD on liver fibrosis by considering the concentrations of components and metabolites, which might be a model for investigation of MoA of other Chinese herbs.

**Supplementary Information:**

The online version contains supplementary material available at 10.1186/s13020-021-00473-8.

## Background

Liver fibrosis is a pathological condition that occurs as a response to chronic liver injury. Various pathological factors, such as hepatitis B and C viruses (HBV and HCV), alcoholic liver disease (ALD), non-alcoholic fatty liver disease (NAFLD) especially non-alcoholic steatohepatitis (NASH), primary biliary cholangitis, primary sclerosing cholangitis, and other autoimmune liver diseases, all contribute to the development of liver fibrosis [1]. After liver injury, the repair process of damaged liver involves two distinct paths: one is the regenerative path, in which injured cells are replaced by the same type of cells; the other is fibrosis, in which normal parenchymal tissue is replaced by connective tissue in an uncontrolled manner [2]. In the context of chronic liver injury, hepatic stellate cells (HSCs) are over-activated, which triggers the excessive deposition of extracellular matrix (ECM) proteins and tissue structural remodelling [3]. Liver fibrosis can further progress to liver cirrhosis, hepatocellular carcinoma, or even death. However, currently there is no validated anti-fibrogenic therapy yet [4–7].

Huangqi decoction (HQD) is a classical formula of traditional Chinese medicine (TCM) to improve liver function and life quality in patients with chronic liver disease. It consists of *Radix Astragali* (*R. Astragali*) and *Radix Glycyrrhizae* (*R. Glycyrrhizae*) in a ratio of 6: 1, so it is also known as Huangqi Liuyi decoction.

*R. Astragali*, known as Huangqi in China, is one of the most commonly used herbal medicines in TCM. It is the dried root of *Astragalus membranaceus* (Fisch.) Bge. or *Astragalus membranaceus* (Fisch.) Bge. var. *mongholicus* (Bge.) Hsiao. As a “Qi tonifier”, *R. Astragali* could invigorate Qi to improve blood circulation and promote the discharge of pus and the growth of new tissue according to TCM theory [8]. Modern pharmacological studies have indicated that *R. Astragali* possesses many biological functions including immunomodulatory, hepatoprotective, anti-hyperglycemic, anti-inflammatory, antioxidant, and antiviral activities, among others [9].

*R. Glycyrrhizae*, Chinese name Gancao, is also one of the most commonly used and oldest herbs in TCM. It is the dried roots and rhizomes of three Glycyrrhiza species—*Glycyrrhiza uralensis* Fisch., *Glycyrrhiza inflata* Bat., and *Glycyrrhiza glabra* L., recorded in Chinese Pharmacopoeia. *R. Glycyrrhizae* has the reputation of “national elders”, which is attributed to its detoxification function to reconcile the toxicity of various herbs [10]. In clinical practice, *R. Glycyrrhizae* has been used to manage various symptoms in different organ systems, such as cough, sore throat, influenza, and liver damage. *R. Glycyrrhizae* has been shown to have antioxidant, anti-inflammatory, anti-viral, anti-diabetic, cytotoxic and cholinergic activities [11].

Researches indicate that HQD exerts significant therapeutic effects on liver fibrosis or cirrhosis induced by dimethyl nitrosamine [12–14]. Modern pharmacological studies have shown that the key mechanisms of HQD in the treatment of liver fibrosis include anti-oxidative stress, inhibition of HSC activation, inhibition of hepatocyte apoptosis and trans-differentiation, inhibiting inflammatory regulation of immunity, inducing hepatic oval cells to differentiate into bile duct epithelial cells, and so on [15].

Most herbal medicines are administered orally. Afterwards, the herb ingredients are directly absorbed into the blood through the digestive tract, or decomposed into secondary metabolites by the action of the intestinal flora to enter the blood, or metabolized into active metabolites by the liver microsomal enzymes. Either way, it can only work if it is transported through the blood to various organs, tissues, or targets and reaches a certain blood concentration. No matter how many ingredients are contained in an herb, only those entering into the blood can become effective ingredients (except external medication and herbs that directly stimulate the gastrointestinal tract). The constituents include prototype components contained in an herb, metabolic products of prototype components, and physiologically active substances.

Traditional research modes of TCM only focus on the effect of herbs on the human body, to blindly study which ingredients are contained in TCM, and which ones show activities in pharmacological experiments in vivo or in vitro. However, the impact of the human body on TCM has always been ignored, which often leads to misunderstanding results. The determining active ingredients may be general constituents, or prodrugs of the active ingredients, and cannot clarify the pharmacodynamics material basis of TCM.

In 2018, Xie et al. published a research work in Clinical Pharmacology & Therapeutics, which studied the process of absorption and metabolism of drug components in healthy volunteers after taking HQD [16]. That work reported the pharmacokinetics of multi-component drugs in vivo for the first time by metabolomics method. It is more scientific and practical than traditional study which only focuses on the original components of herbal medicines and ignores the influence of metabolism. However, the authors only discussed the possible effects of several active components in *R. Astragali* and *R. Glycyrrhizae*, which have been studied and reported many times. They did not elucidate the specific MoA of HQD on liver fibrosis at the target level.

In this study, taking advantage of Xie’s metabolomics data, we applied network pharmacology and molecular docking methods to reveal principal components of HQD, the MoA of the components and their metabolites in vivo against liver fibrosis.

## Methods

### Data collection and preparation

At first, we collected prototype compounds of HQD and their metabolites from the supporting information of Xie’s paper [16]. The screening rules for compounds were listed as following: (1) Compounds whose structural information could not be obtained by chemical names were deleted. (2) Compounds whose 16 Fc (fold change) values were less than 2 were deleted (for each compound, Xie’s paper provided the Fc value for 16 time periods), the reason for this rule is because the concentrations of some compounds were not changed significantly after administration, and such compounds were not considered to be effective. (3) We also deleted those with short aliphatic chains, and those usually not considered as the major active compounds according to literature, such as various amino acids and their derivatives. In addition, we deleted such simple compounds as hydroquinone and 1,4-Dithiothreitol. Compounds that meet the above screening criteria were retained, and their structures were downloaded from the NCBI PubChem (http://www.ncbi.nlm.nih.gov/pccompound/), which were saved as sdf format.

To determine which herb the prototype compounds came from, *R. Astragali* or *R. Glycyrrhizae*, we did a literature search using PubMed (https://pubmed.ncbi.nlm.nih.gov/), Web of Science (http://apps.webofknowledge.com) and CNKI (http://www.cnki.net/). The compounds were divided into three classes: (1) compounds from *R. Astragali*, (2) compounds from *R. Glycyrrhizae*, and (3) common compounds from both. It should be noted that for those present in both herbs, if there are concentration data of the compounds in one herb rather than the other, the compounds are considered to belong to that herb with concentration data.

The known compound-target interactions (CTIs) were collected from PubChem [17], IUPHAR/BPS Guide to PHARMACOLOGY [18], PharmGKB [19], BindingDB [20], and DrugBank [21]. An interaction between a compound and a protein target was defined by K_i_, K_d_, IC_50_, or EC_50_ ≤ 10 μM. Duplicates were removed.

The live fibrosis-related genes were obtained from eight gene-disease databases, including GEO [22], Diseases [23], GeneCards [24], OMIM [25], PharmGKB [19], TTD [26], DisGeNET [27], and MalaCards [28], with key words “liver fibrosis”, or “hepatic fibrosis”, or “hepatitis B virus”, or “hepatitis C virus”, or “non-alcoholic fatty liver disease”, or “non-alcoholic steatohepatitis”, or “alcohol abuse”, or “alcoholism”, or “alcoholic hepatitis”, or “fatty liver”, or “primary biliary cholangitis”, or “primary sclerosing cholangitis”, or “autoimmune hepatitis”, or “hemochromatosis”.

### Prediction of targets and construction of compound-target interaction network

Potential targets of compounds were predicted using our own web server NetInfer (http://lmmd.ecust.edu.cn/netinfer/) [29]. The balanced substructure-drug-target network-based inference (bSDTNBI) method and the global drug-target interaction network (version 2016) were selected. The molecular fingerprint was set to Klekota-Roth, and other parameters were set as default values. For each compound, the top 20 predicted targets were obtained. Some compounds have known targets, which were also provided on the target list. Finally, these targets were normalized to the official gene name using the UniProt database (https://www.uniprot.org/).

To construct the global compound-liver fibrosis target (CLFT) bipartite network, firstly, the known and predicted targets of all the compounds were brought together. Then, these targets were mapped into the liver fibrosis-related genes, and the overlapped targets were saved as HQD-liver fibrosis targets. Before we constructed the network, compounds were labelled according to their herb belongings. Finally, the bipartite network was constructed via Cytoscape.

### Construction of protein–protein interaction network and selection of hub genes

**Fig. 1:**
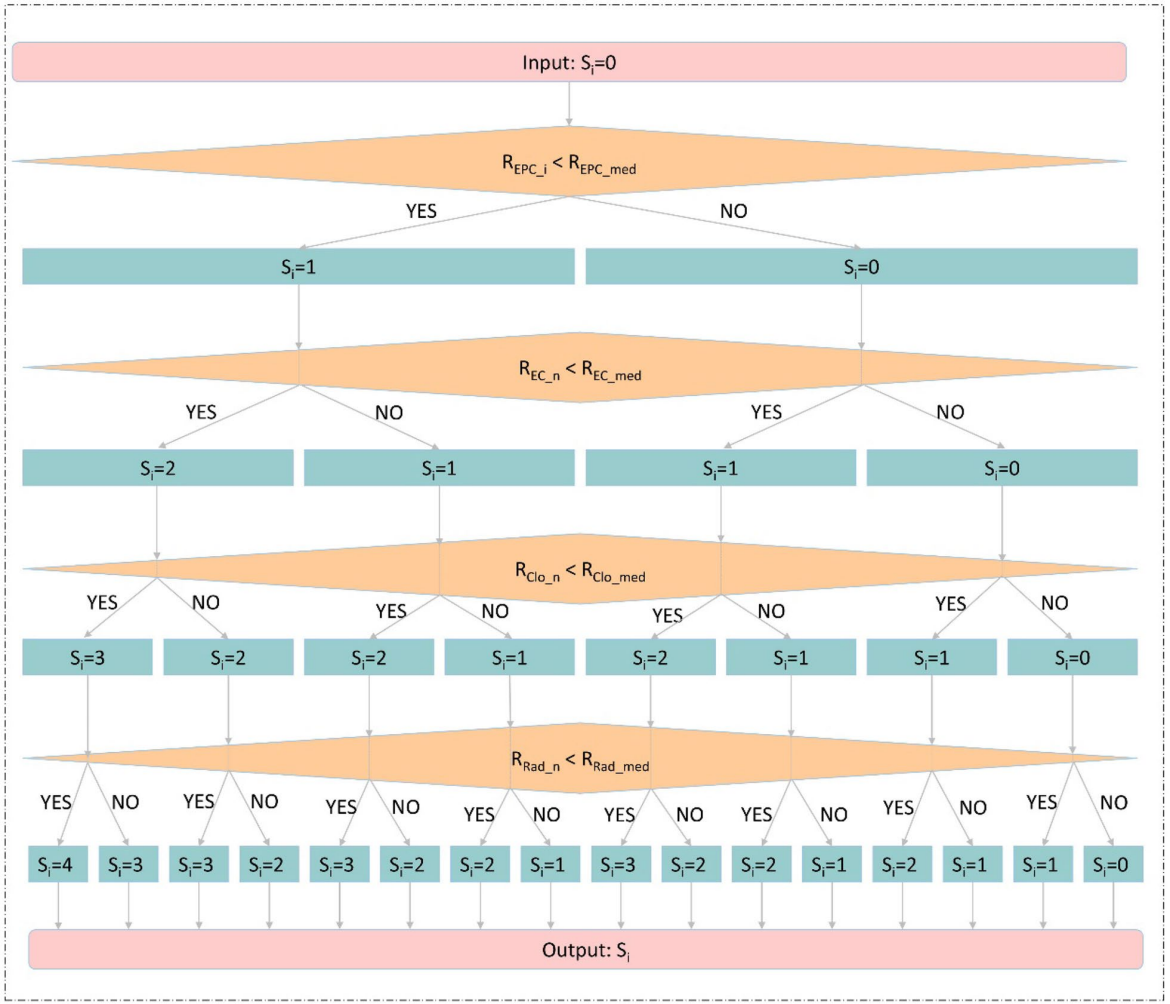
The calculation process of scores of targets. *S*_*i*_ refers to the score of the i-th target; *R*_*EPC_i*_ refers to the ranking of the i-th target in EPC item, *R*_*EPC_med*_ refers to the median value of the ranking of all targets in this item; *R*_*EC_i*_ refers to the ranking of the i-th target in EC item, *R*_*EC_med*_ refers to the median value of the ranking of all targets in this item; *R*_*Clo_i*_ refers to the ranking of the i-th target in Clo item, *R*_*Clo_med*_ refers to the median value of the ranking of all targets in this item; *R*_*Rad_i*_ refers to the ranking of the i-th target in Rad item, *R*_*Rad_med*_ refers to the median value of the ranking of all targets in this item

Targets that in the global CLFT bipartite network were uploaded to Cytoscape to identify the interactions between them by BisoGenet [30]. The obtained protein–protein interactions (PPIs) were further analyzed by cytoHubba [31]. Four node-ranking methods, including Edge Percolated Component (EPC), EcCentricity (EC), Closeness (Clo), and Radiality (Rad), were used to select hub nodes in the PPI network. For each method, it gave a score for each node, and we gave a ranking based on each score. The higher is the score, the higher the ranking is. For example, the node with the highest score is ranked first. Nodes with the same score were ranked the same, no matter how many nodes with the same ranking, the next node's ranking will only increase by one. After that, each node had four rankings according to the four methods. Finally, we calculated scores for nodes according to their four rankings as following:
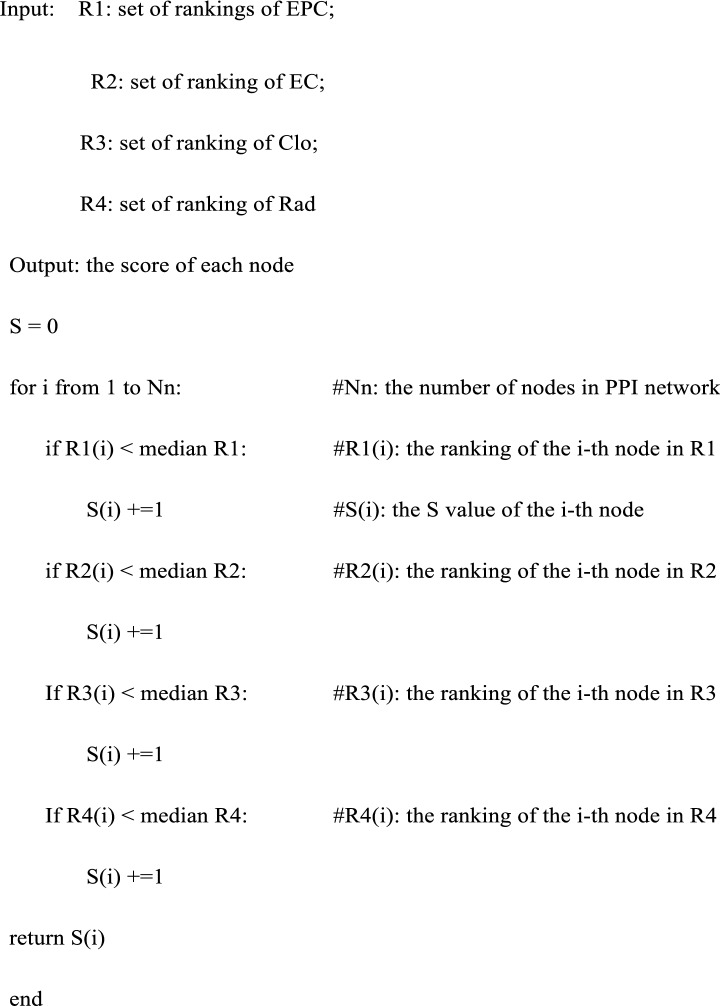


As shown in Fig. 1, for the i-th target, its initial score (S_i_) is 0. In the EPC item, if its ranking is less than the median value of the rankings of all targets in this item, the score of the i-th target is increased by 1. The same is for EC, Clo, and Rad items. For these targets with a final score greater than 1, if there was more than one compound interacting with them, then they were selected as hub genes.

### Enrichment of KEGG pathways

Hub genes were imported to STRING (https://string-db.org/) [32] database to perform KEGG pathway enrichment analysis. Only KEGG pathways with the false discovery rate < 0.05 were regarded as significant and were retained. Based on the knowledge accumulated in previous literature research, we deleted pathways that were less related to liver fibrosis, such as cancer-related pathways, and only those closely related to liver fibrosis were retained.

### Molecular docking and identification of critical compound-target interactions

Molecular docking was performed to evaluate the potential interactions between critical compounds and liver fibrosis targets by the Glide module of Schrodinger’s Maestro molecular modeling suite (Schrödinger Release 2019-2). We collected all the crystal structures of hub gene-encoded proteins that appeared in retained pathways closely related to liver fibrosis from the Protein Data Bank (PDB, https://www.rcsb.org/) [33]. Only those with relatively higher resolution were reserved for molecular docking.

The protein preparation module of Maestro that named Protein Preparation Wizard was used to prepare the protein crystallographic structures. Water molecules were deleted from the structures, and the amide moieties in the side chain were adjusted to optimize their interactions with surrounding residues and groups of atoms. The centroid of the co-crystalized ligand in the crystal structures of complex was defined as the binding site. The proteins were then minimized using the OPLS_2005 force field with a default constraint of 0.30 Å root-mean-square deviation. The three-dimensional structures of compounds were generated using the LigPrep module in Maestro. OPLS_2005 force field was also used; the “Ionization” option was set to “Neutralize”; the “Computation” option selected the second item that named “Determine chiralities from 3D structure”. After the ligand grid was generated and the compounds were prepared, the SP (standard precision) mode of Ligand Docking module in Maestro with the default settings was performed to dock.

The Glide Gscore greater than the median value of all the Glide Gscores indicated a potential strong binding ability of candidate targets to their corresponding compounds. The docking results were visualized by PyMOL software, and the hydrogen bonds and their binding sites were observed and analyzed.

Before we constructed the critical CLFT bipartite network, the critical CTIs need to be identified from known and predicted CTIs that have been docked, and the selection criteria were as following: (1) For known CTIs of each target, the one with larger FC_MAX value of the compound was reserved. (2) For predicted CTIs of each target, the one CTI with lower Gscore and greater the FC_MAX values of the compound was retained. In addition, we performed cross docking between targets and compounds in the selected predicted CTIs. After that, the obtained predicted and known CTIs were imported to Cytoscape to construct the critical CLFT bipartite network.

## Results

### Data collection

The original prototype components of HQD and their metabolites were collected from the supporting information of Xie’s paper [16]. After refinement via a few screening criteria, a total of 68 compounds, including 17 prototype components and 51 metabolites, were finally obtained.

To further clarify the relationships between compounds and genes, according to the division rules, we realized that four of the 17 prototype compounds were derived from *R. Astragali*, eight from *R. Glycyrrhizae*, and five from both herbs. As for most of the metabolites that cannot be identified as herb belongings from literature, we did not classify them. Details of the 68 compounds were listed in Additional file 1: Table S1.

We also tried to search for the known targets for the 68 compounds from four databases, including PubChem [17], IUPHAR/BPS Guide to PHARMACOLOGY [18], PharmGKB [19], BindingDB [20], and DrugBank [21]. After removing duplicates, 186 known CTIs were obtained for only 23 of the 68 compounds.

Liver fibrosis is not a single disease but a pathological concept for characterizing a variety of chronic liver diseases, so we regarded those genes related to several types of chronic liver diseases to cause liver fibrosis as liver fibrosis-related genes. Then eight gene-disease databases, including GEO [22], Diseases [23], GeneCards [24], OMIM [25], PharmGKB [19], TTD [26], DisGeNET [27], and MalaCards [28], were searched with a lot of key words. After removing the duplicates, 1192 liver fibrosis-related genes were obtained.

### Compound-liver fibrosis target bipartite network

A compound-target bipartite network is helpful for understanding the molecular mechanism of chemical action. However, only 23 of the 68 components in HQD were found to have known targets, and most of them do not have. Therefore, potential targets were predicted for all the 68 compounds by our web server NetInfer [29], and 1360 CTIs were acquired. Combining with the above 186 known CTIs, totally 1520 CTIs were obtained after deleting duplicates, which were involved in 310 targets (see Additional file 2: Table S2).

**Fig. 2:**
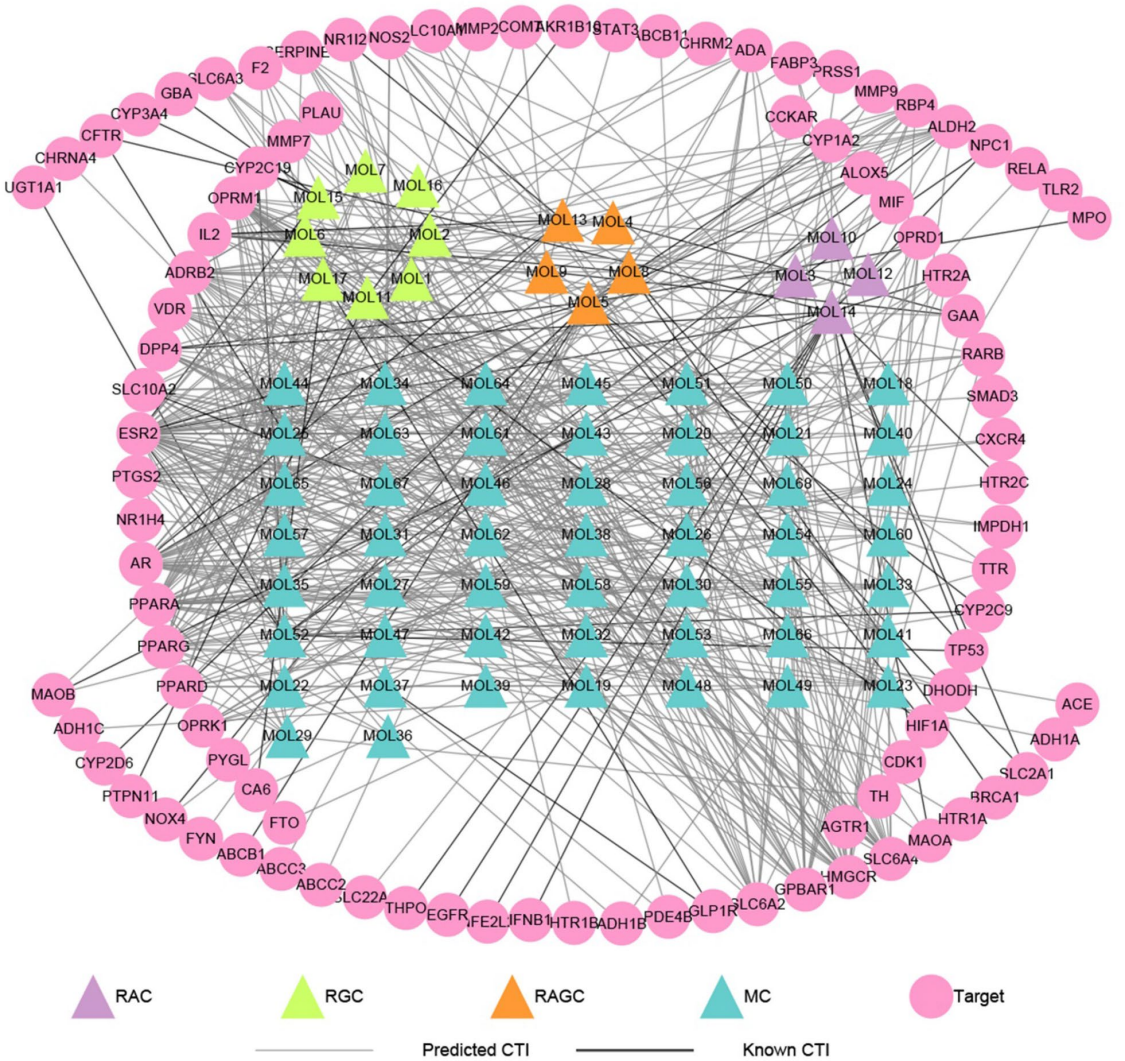
The global bipartite network between chemical components and liver fibrosis genes. Pink circle nodes represent potential targets, triangle nodes remark chemical components and each edge stands for an interaction between them. Purple triangle nodes: components from *R. Astragali* (RAC); green triangle nodes: components from *R. Glycyrrhizae* (RGC); orange triangle nodes: components from both (RAGC); blue triangle nodes: metabolic products (MCs). Silver edges: predicted CTIs; black edges: known CTIs

Among the 310 targets, 95 ones were present in the collected 1192 liver fibrosis-related genes, which led to 540 CTIs between the 95 targets and the 68 components. A global compound-liver fibrosis target (CLFT) bipartite network was then constructed via Cytoscape 3.8.0, as shown in Fig. 2. In total, this bipartite network consisted of 163 nodes and 540 edges, with 68 compounds as triangle nodes and 95 targets as circle nodes.

To more intuitively represent the relationships between compounds and targets, 68 compounds were divided into four groups according to the division rules: four from *R. Astragali* (colored with purple), eight from *R. Glycyrrhizae* (colored with green), five from both herbs (orange), and the other 51 metabolites (blue). It is obvious that the number of compounds in *R. Astragali* is less than that in *R. Glycyrrhizae*. Among these compounds, Genistein (MOL14), Kaempferol (MOL5), Glycyrrhetinic acid (MOL11), Apocholic acid (MOL48), Daidzein (MOL52), Hyocholic acid (MOL55), and Lucidenic acid G (MOL59) have the highest number of targets. The 540 CTIs include 53 known CTIs and 487 predicted CTIs. Among the 53 known CTIs, Genistein has interactions with 14 targets, Kaempferol has associations with 12 targets, both compounds are well-studied in herbal medicine.

### Protein–protein interaction network and KEGG pathways

**Fig. 3:**
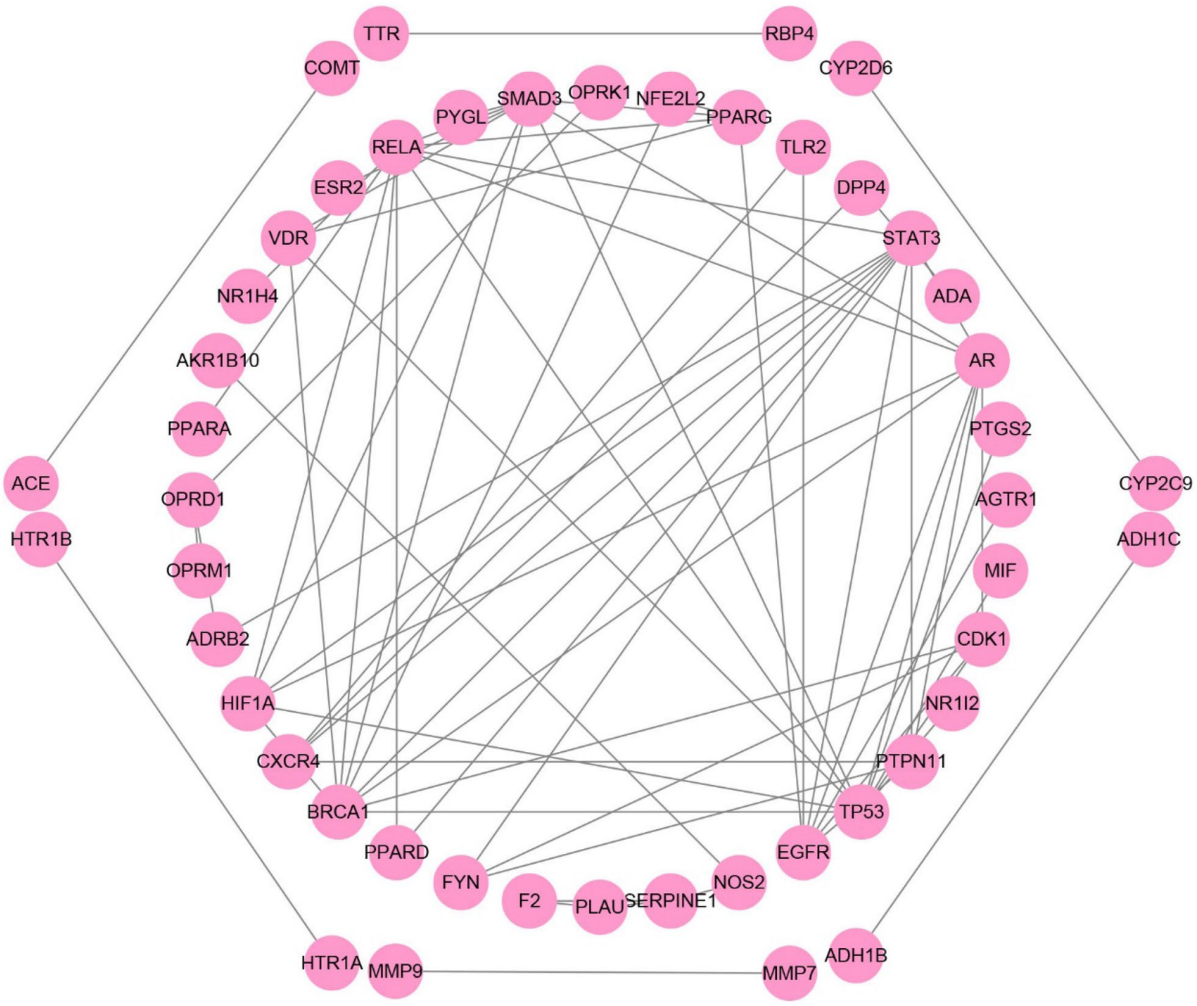
HQD-liver fibrosis protein–protein interaction network

Besides the direct interactions with target proteins, the compounds might also affect the other proteins indirectly, for example, via protein–protein interactions (PPIs). Therefore, PPIs were searched for the 95 targets in the global CLFT bipartite network via BisoGenet, a Cytoscape plugin [30], which resulted in a total of 71 PPIs for 48 of the 95 targets. Then, the HQD-liver fibrosis PPI network was constructed, as shown in Fig. 3.

In order to identify important targets from the PPI network, four values including Edge Percolated Component (EPC), EcCentricity (EC), Closeness (Clo), and Radiality (Rad) were calculated by cytoHubba, another Cytoscape plugin [31] for each node in the PPI network. After calculation, there were seven targets, including AR, PPARG, CDK1, TP53, HIF1A, VDR, and PPARD with a final score greater than 1, and more than one compound has interactions with them. Although PTGS2, SERPINE1 and MMP9 did not meet the requirements, they have also opted for the next step based on the knowledge of previous literature research.

**Fig. 4:**
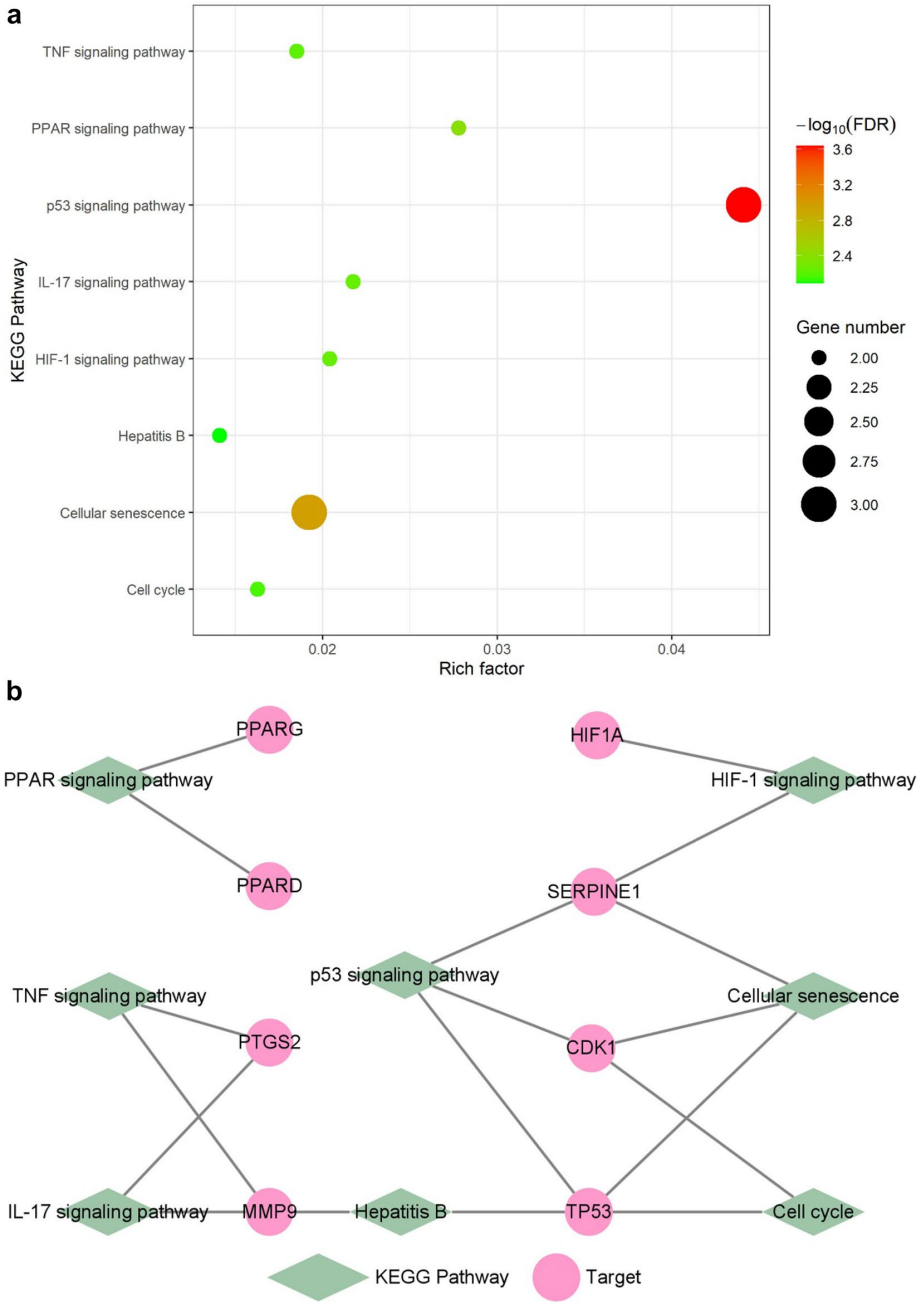
KEGG pathways. **a** Dot plot of the eight KEGG pathways. **b** The pathway-gene bipartite network. Green diamond nodes remark KEGG pathway, pink circle nodes remark target

These 10 hub genes were enriched into 30 KEGG pathways with significance. Except for cancer-related pathways (e.g. Pathways in cancer (hsa05200), Prostate cancer (hsa05215), etc.) and other pathways (for instance, Longevity regulating pathway (hsa04211), etc.) that were not directly related to liver fibrosis based on knowledge from literature research, eight liver fibrosis-related pathways were selected for further analysis, as shown in Fig. 4a.

These eight pathways include p53 signaling pathway (hsa04115), Cellular senescence (hsa04218), PPAR signaling pathway (hsa03320), HIF-1 signaling pathway (hsa04066), IL-17 signaling pathway (hsa04657), TNF signaling pathway (hsa04668), Cell cycle (hsa04110), and Hepatitis B (hsa05161). CDK1, SERPINE1, and TP53 were enriched in p53 signaling pathway and Cellular senescence, which were related to cell cycle arrest according to the KEGG Pathway (https://www.kegg.jp/kegg/pathway.html). Furthermore, peroxisome proliferator-activated receptor (PPAR) family genes, including PPARD and PPARG were enriched into one pathway—PPAR signaling pathway, which is mainly related to liver lipid metabolism. The HIF-1 signaling pathway contains HIF1A and SERPINE1 and is related to angiogenesis. MMP9 and PTGS2 were enriched into IL-17 signaling pathway and TNF signaling pathway, two inflammation-related pathways. Besides, Hepatitis B also appeared, which was pathogenic factor of liver fibrosis. The pathway-gene bipartite network was shown in Fig. 4b, consisting of eight pathways and eight genes, and each pathway contains two or three genes.

### Molecular docking to evaluate critical compound-target interactions

There were eight genes in the above eight pathways, and 43 compounds have interactions with these eight targets in the global CLFT bipartite network, of which 23 compounds interacted with PPARG, 17 compounds had links with PTGS2, 10 compounds could act on PPARD, and 7 compounds were associated with SERPINE1. For the convenience of analyzing the relationships between critical compounds and targets from the compounds that could act on PPARG, PTGS2, PPARD, or SERPINE1, we selected some compounds that have a higher concentration in vivo after administration of HQD. Concretely speaking, for metabolites that have interactions with PPARG or PTGS2, only the maximum of fold change (FC_MAX) values greater than 10 were reserved; while for metabolites that could act on PPARD or SERPINE1, compounds with a maximum value of FC greater than 5 remained. Finally, 34 CTIs (including 5 known CTIs and 29 predicted CTIs) were reserved, including eight targets and 27 compounds.

We performed molecular docking on these 29 predicted CTIs, including six targets (CDK1, MMP9, PPARD, PPARG, PTGS2, and SERPINE1) and 23 compounds (see Additional file 3: Table S3). Notably, the lower of Glide Gscore meant the binding between the compounds and the targets were stronger. Then, according to the above selection criteria, finally, seven predicted CTIs and three known CTIs were regarded as critical CTIs, which include eight compounds and eight targets.

**Fig. 5:**
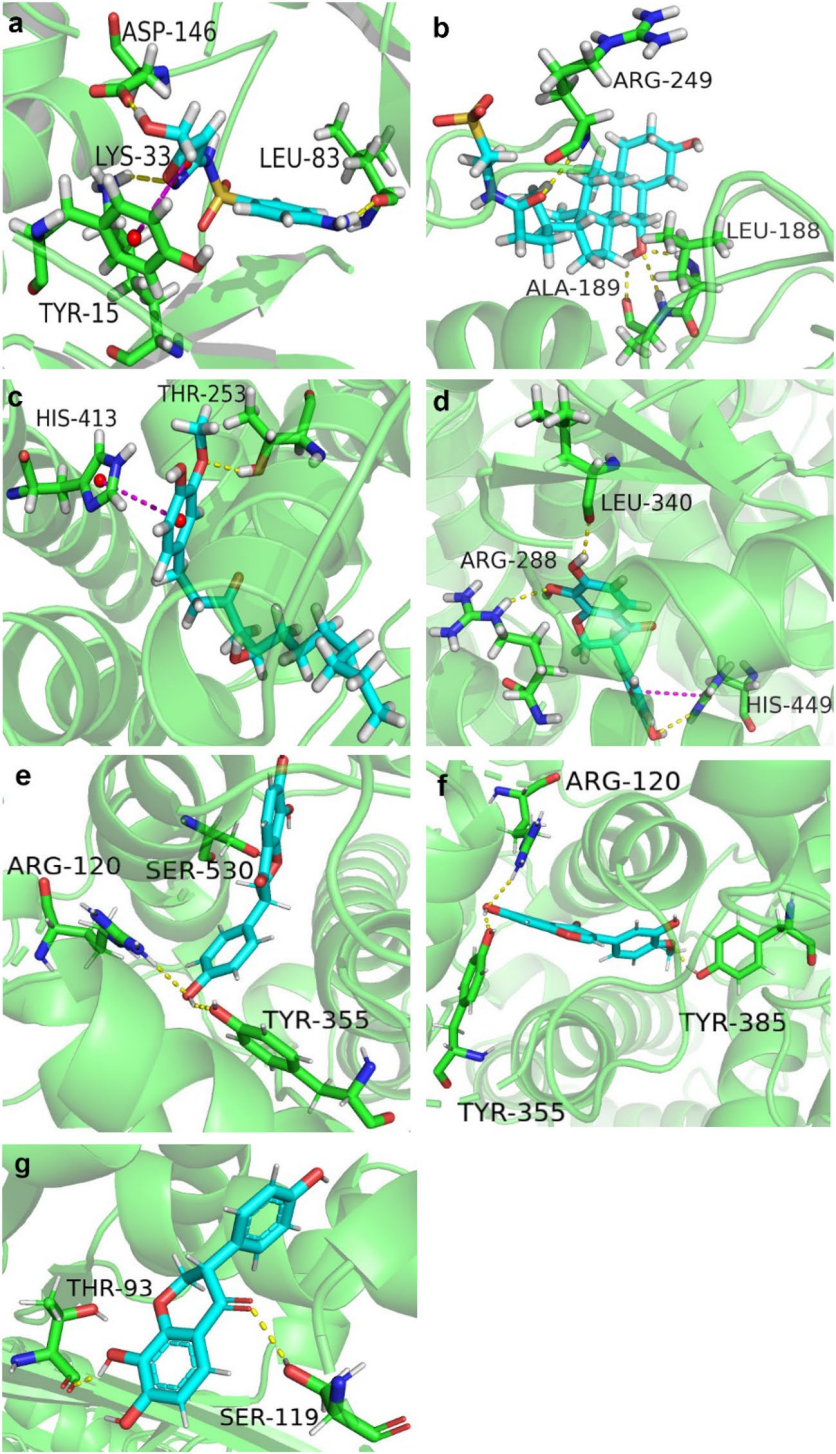
The interaction models of seven predicted CTIs. **a** MOL39 with CDK1, **b** MOL67 with MMP9, **c** MOL18 with PPARD, **d** MOL43 with PPARG, **e** MOL43 with PTGS2, **f** MOL10 with PTGS2, **g** MOL43 with SERPINE1

The Gscore values of seven predicted CTIs were listed in Table 1. As shown, 4′,7,8-Trihydroxyisoflavanone (MOL43) had strong interactions with three targets, namely PTGS2, PPARG and SERPINE1, with high scores of − 8.253, − 7.534 and − 6.667, respectively. The binding sites of the seven predicted CTIs were shown in Fig. 5. According to Fig. 5 and Table 1, all the five compounds in the seven interaction models demonstrated good bindings with the six hub genes, suggesting that HQD had a strong tendency as a therapeutic strategy for liver fibrosis via these hub genes and compounds. As shown, there were several strong bindings, including 5-Hydroxysulfamethoxazole (MOL39) with CDK1 (Gscore = − 6.125), Tauroursodeoxycholic acid (MOL67) with MMP9 (Gscore = − 6.906), (S)- [8]-Gingerol (MOL18) with PPARD (Gscore = − 7.786), 4′,7,8-Trihydroxyisoflavanone (MOL43) with PPARG (Gscore = − 7.534), 4′,7,8-Trihydroxyisoflavanone (MOL43) with PTGS2 (Gscore = − 8.253), Calycosin (MOL10) with PTGS2 (Gscore = − 8.29), 4′,7,8-Trihydroxyisoflavanone (MOL43) with SERPINE1 (Gscore = − 6.667). The Gscore values of cross docking were shown in Additional file 4: Table S4. From the results of cross docking, it is easy to see that the docking results roughly matched the predicted results in general, only slight differences in some numerical values, which is reasonable. For PPARG and PTGS2, the predicted CTIs are the highest scores. For PPARD and SERPINE1, the scores of predicted CTIs are slightly lower than scores for MOL10 with the targets. As for CDK1 and MMP9, the scores of two predicted CTIs were not the highest, this may be due to the complex structures of the two compounds.

**Table 1:** The Glide Gscores of seven PCTIs

Compound ID	Compound name	Target	Glide Gscore
MOL39	5-Hydroxysulfamethoxazole	CDK1	− 6.125
MOL67	Tauroursodeoxycholic acid	MMP9	− 6.906
MOL18	(S)-[8]-Gingerol	PPARD	− 7.786
MOL43	4′,7,8-Trihydroxyisoflavanone	PPARG	− 7.534
MOL43	4′,7,8-Trihydroxyisoflavanone	PTGS2	− 8.253
MOL43	4′,7,8-Trihydroxyisoflavanone	SERPINE1	− 6.667
MOL10	Calycosin	PTGS2	− 8.29

The interaction model of MOL39 in the active site of CDK1 (Fig. 5a) showed the presence of a pi-pi stacking with the key residue Tyr15, in addition to the formation of three hydrogen bonds with residues Asp146, Leu83, Lys33, which may help the stabilization of the ligand in the active site of the target protein. Four hydrogen bonds were observed between MOL67 and residues Leu188, Ala189 and Arg249 in the active site of MMP9 (Fig. 5b). MOL18 showed favorable binding with PPARD, where its interaction diagram in the binding site of PPARD (Fig. 5c) showed the formation of a stacking pi–pi interaction between the aromatic ring of MOL18 and residue His413, a hydrogen bond was observed between MOL18 and Thr253. The interaction diagram between MOL43 and PPARG (Fig. 5d) showed the formation of a pi-pi stacking with the key residue His449, and the formation of three hydrogen bonds with residues His449, Leu340 and Arg288. Likewise, MOL43 showed a high affinity towards PTGS2 (Fig. 5e). Hydrogen bonds was also observed between MOL43 and Arg120, Ser530, Tyr355. Besides, among all the predicted CTIs, MOL43 showed the lowest Gscore against SERPINE1 (Fig. 5g), it only formed two hydrogen bonds with residue Thr93 and Ser119. The interaction diagram between MOL10 and PTGS2 (Fig. 5f) showed the formation of four hydrogen bonds with residues Tyr355, Arg120 and Tyr385.

### Critical compound-target interaction network and mechanism analysis

**Fig. 6:**
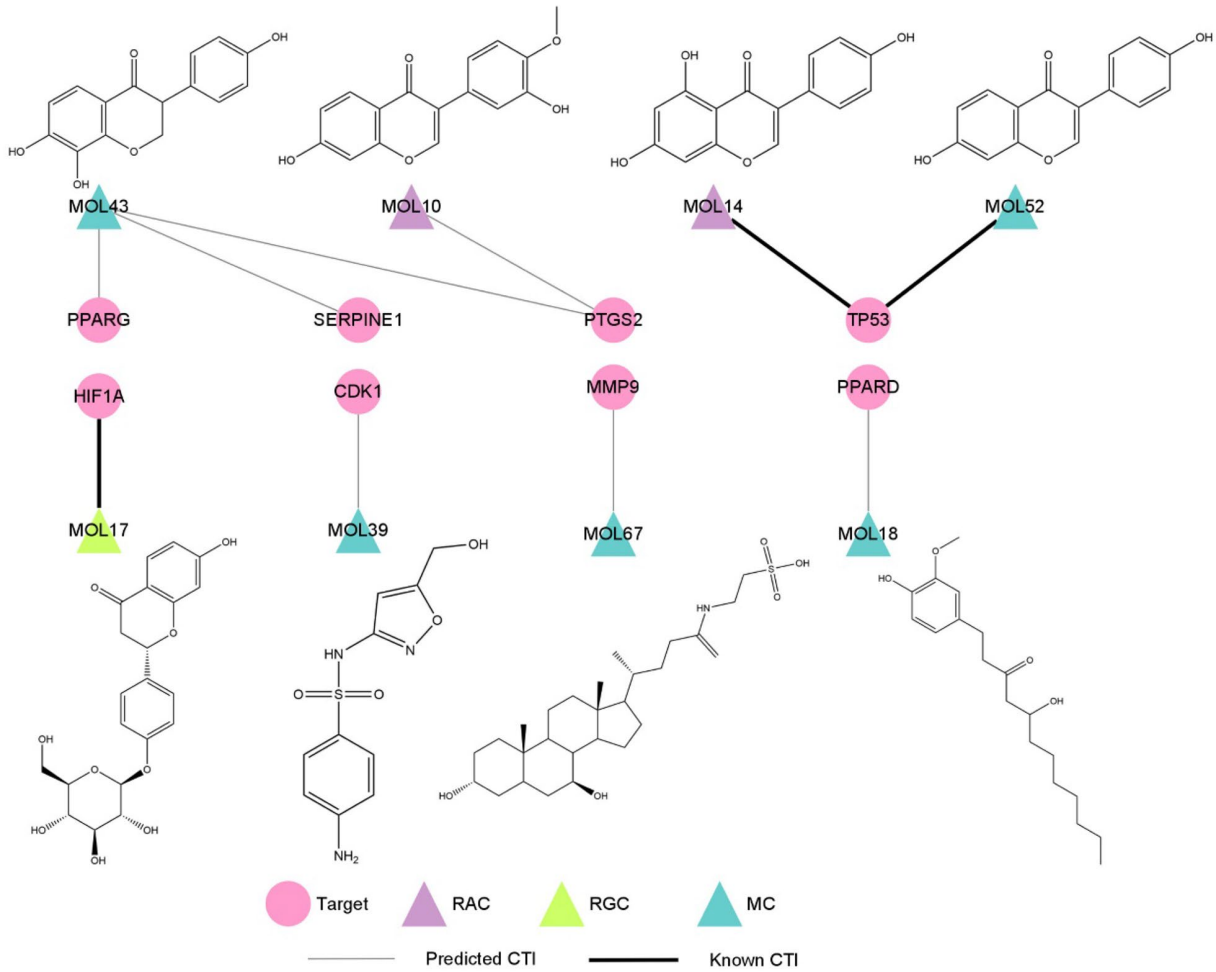
The bipartite network between critical components and liver fibrosis genes. Similar to Fig. 1, pink circle nodes: critical targets, circle nodes: critical components. Purple nodes: components from *R. Astragali* (RAC), green triangle nodes: components from *R. Glycyrrhizae* (RGC), blue triangle nodes: metabolic products (MCs). Silver edges: predicted CTIs, black edges: known CTIs

According to the results of molecular docking and chemical concentration, seven predicted CTIs and three known CTIs constructed the critical CLFT bipartite network, as shown in Fig. 6. In the graph, there were two compounds from *R. Astragali* (colored with purple), one from *R. Glycyrrhizae* (green) and five metabolites (blue). Among them, MOL10, MOL14, MOL17, MOL43 and MOL52 were flavonoids. In addition, three known CTIs (black edge) were MOL14 and MOL52 targeting TP53, MOL17 acting on HIF1A. Seven predicted CTIs (silver edge) include MOL43 to PTGS2, PPARG and SERPINE1, respectively; MOL10 to PTGS2; MOL39 to CDK1; MOL67 to MMP9; MOL18 to PPARD. It can be found that except for MOL43 which interacted with three targets, all other compounds were directed against a single target. Among the eight critical compounds, MOL43, a metabolite compound, has the highest concentration in vivo after administration of HQD with FC_MAX = 2390.64, followed by MOL10 and MOL17, with FC_MAX = 772.69 and 14.35, respectively. It is worth noting that MOL10 is a prototype compound in *R. Astragali* and MOL17 is an original component of *R. Glycyrrhizae*. Furthermore, from the structures of these eight compounds, it is easy to see that MOL10 and MOL14, two prototype compounds in *R. Astragali*, have similar structures, in addition, MOL43 and MOL52, two metabolites, have similar structures with MOL10 and MOL14. For this reason, we can reasonably speculate that MOL43 and MOL52 may be metabolites of original components in *R. Astragali*.

## Discussion

As a classical herb pair, HQD has been used to improve liver function and quality of life in patients with chronic liver disease, such as liver fibrosis [34]. Though there have been some studies on HQD [12, 13, 35, 36], there is a lack of target-level study on the mechanisms of its prototype compounds and their metabolites in the treatment of liver fibrosis. Since the significance of metabolites in Chinese medicine is gradually attracting attention, here, we tried to understand the MoA of HQD from a systematic perspective by combining metabolomics data with network pharmacology and molecular docking methods. According to the results of KEGG pathway analysis, network topology analysis and molecular docking simulation, we analyzed the potential MoA for HQD to treat liver fibrosis through eight critical compounds (three prototype compounds and five metabolites) and eight critical targets.

MMP9, HIF1A and SERPINE1, three targets that in the critical CLFT bipartite network, in addition to MMP2, a target not appeared in the critical CLFT network but in the global CLFT network, which were correlated with fibrogenesis and degradation. Liver fibrosis is a dynamic pathologic process characterized by an accumulation of the ECM, which is a consequence of an imbalance between ECM deposition and degradation, reflecting dysregulation of matrix metalloproteinases (MMPs) and their specific inhibitors (tissue inhibitors of metalloproteinases, TIMP) [37]. Upon chronic damage of liver tissue, HSCs become activated and differentiate into a fibroblast-like phenotype, and upregulated the expression of TIMP1, which leading to the inhibition of MMP activity and subsequent accumulation of ECM [37]. In the family of MMPs, MMP2 and MMP9 are particularly important for the development of liver fibrosis since they degrade type IV collagen (basal membrane) [38]. However, some studies also demonstrated MMPs, especially MMP2 and MMP9, promoted HSCs proliferation and migration [39, 40]. There were some literatures about MMP2 and MMP9 related to liver fibrosis, such as MMP9 was up-expressed in HCV patients with different stages of fibrosis [41], the activity of MMP2 and MMP9 in patients with liver cirrhosis were increased [42] and so on. There was also a trend for higher serum MMP9 in patients with HCC [43]. In contrast, it also has been reported that MMP2 and MMP9 levels showed a significant elevation in chronic HCV patients [44]. Several studies have shown that hypoxia-inducible factor-1α (HIF1A) is critical for upregulation of pro-fibrotic mediators, such as platelet-derived growth factor A/B, and plasminogen activator inhibitor-1 (SERPINE1), and mice deficient in HIF1A had reduced liver fibrosis [45–47]. Moreover, MMP2 has been proved to be positively correlated with HIF1A protein levels in HCC tissues, the expression levels of MMP2 and HIF1A in the HCC tissues were higher than those in the adjacent normal tissues [48]. Plasma SERPINE1 level was significantly increased in children with increased severity of steatosis, and fibrosis [49]. Higher expression of SERPINE1 was also found to be present in adults with NAFLD and children with NASH [50, 51]. SERPINE1 deficiency reduced cholestatic liver injury and fibrosis [52, 53]. Wang et al. also found that SERPINE1 deficiency reduced hepatic fibrosis after bile duct obstruction [54].

Some compounds in the global CLFT network have been reported to inhibit the expression of MMP2, or MMP9, or HIF1A, or SERPINE1. Liang et al. reported that expression of MMP2 and MMP9 proteins were up-regulated in carbon tetrachloride-induced liver injury, while treatment with MOL2 significantly reduced the expression levels of MMP2 and MMP9 proteins [55]. MOL7 and MOL14 were found to decrease MMP2 [56, 57]. MOL7, MOL10, MOL17 and MOL9 were reported to down-regulate MMP9 [58–60]. MOL1, MOL5, MOL12, MOL13, MOL15, and MOL52 showed reduction in MMP2 and MMP9 expression level [61–66]. As for HIF1A, there were seven compounds were reported to suppress the expression of HIF1A, including MOL5, MOL6, MOL9, MOL10, MOL13, MOL14, and MOL15 [67–73]. Lee et al. found that MOL7 significantly inhibited liver fibrosis through blocking the transforming growth factor-β1-induced the transcript levels of SERPINE1 and matrix MMP2 [56]. MOL10 can also exert SERPINE1 inhibitory activity [74]. Based on the above analysis, we can reasonably speculate that 13 compounds (MOL1, MOL2, MOL5, MOL6, MOL7, MOL9, MOL10, MOL12, MOL13, MOL14, MOL15, MOL17, and MOL52) of HQD maintain the balance of ECM and reduces liver damage by regulating the expression of MMP2, MMP9, HIF1A and SERPINE1 to exert the effect of anti-liver fibrosis.

CDK1 and TP53, the other two of the eight critical targets obtained in this study, were enriched in the p53 signaling pathway and cellular senescence signaling pathway, which were related to apoptosis and cell cycle arrest. It has been reported that CDK1 was significantly up-regulated in 309 HCC tissues compared with adjacent tissues [75]. Zhang et al. reported that downregulated cyclin B1 and CDK1, induced caspase-dependent apoptosis, and reduced migration in HSCs [76]. A growing amount of evidence suggests that TP53 performs a central function in the development of chronic liver diseases. For example, Derdak et al. found that inhibition of TP53 attenuated steatosis and liver injury in a NAFLD model [77]. Yahagi et al. demonstrated that TP53 was activated in hepatic steatosis models and the p53 pathway was involved in the pathogenesis of the fatty liver disease [78]. Moreover, hepatocyte apoptosis was linked to TP53 activation in experimental NASH [79]. Based on these findings, overexpression of CDK1 and TP53 may exacerbate liver fibrogenesis. There were four compounds (MOL21, MOL26, MOL39, MOL67) linking to CDK1, and three compounds (MOL8, MOL14, MOL52) interacting with TP53 in the global CLFT bipartite network. Among them, MOL67 was reported to inhibit expression and acetylation of NF-κB and TP53, and attenuated hemorrhagic shock-induced liver injury [80]. For this, compounds of HQD perhaps inhibit hepatocyte apoptosis through regulating the expression level of CDK1 and TP53, and ultimately slow down liver fibrogenesis.

PTGS2, PPARD, and PPARG were the remaining three targets in the critical CLFT network. After hepatocyte injury, inflammation and the activation of the innate immune system lead to HSCs activation and ECM secretion and deposition, which cause liver fibrogenesis [81]. Patients with chronic hepatitis B had significantly higher PTGS2 expression compared with controls [82]. PPARG plays an important role in the inhibition of HSC activation and has been proposed as a potential molecular target for liver fibrosis [83]. There has been clear evidence that PPARG level and activity are reduced in activated HSCs [84]. Activation of PPARG modulates profibrogenic and pro-inflammatory actions in HSCs [80]. Moreover, liver inflammatory responses were also suppressed by PPARA, PPARD and PPAG by inhibition of NF-κB [85]. MOL2 alleviated carbon tetrachloride-induced liver injury partly due to downregulate the expression of pro-inflammatory mediators, including PTGS2 [86]. MOL10 was identified as a PTGS2 inhibitor by using ultrafiltration, enzyme-immobilized magnetic beads, high-performance liquid chromatography, and electrospray-ionization mass spectrometry [87]. In addition, it was reported that MOL10 treatment significantly reduced the overexpression of PTGS2 mRNA [88]. MOL1 was found to attenuate pro-inflammatory cytokines through activating PPARG [89]. MOL7, MOL13, MOL15, and MOL16, also showed an effect on PPARG activation [90]. Moreover, MOL15 has been reported to induce PPARG expression at the protein level and inhibit the expression of PTGS2 [91]. Accordingly, these compounds of HQD may prevent the further development of liver fibrosis by alleviating liver inflammation through influencing the expression of PTGS2, PPARD, and PPARG.

In this study, we visualized an intricate network among prototype compounds and metabolites of HQD and their potential targets of liver fibrosis. Based on our topology analysis and molecular docking simulation, eight compounds (MOL39, MOL67, MOL18, MOL43, MOL10, MOL14, MOL52, and MOL17) and eight targets (CDK1, MMP9, PPARD, PPARG, PTGS2, SERPINE1, TP53, and HIF1A) were regarded as critical compounds and targets for the mechanism of HQD in the treatment of liver fibrosis. The therapeutic effect of HQD on liver fibrosis is mainly attributed to compounds in HQD, which regulate the expression levels of the eight targets to maintain the balance of ECM, reduces liver damage, inhibit hepatocyte apoptosis, and alleviate liver inflammation.

In our research results, not only three prototype compounds (namely MOL10, MOL14 and MOL17) were found to be closely related to the therapeutic effects of HQD, which is consistent with the results of existing experimental studies, but also some metabolites (such as MOL39 and MOL43) have been found to be the key to MoA of HQD on liver fibrosis. Though more biological evidence is needed to further validate the current results, the MoA of HQD in the treatment of liver fibrosis was explored from the target level in a systemic mode by combing the network pharmacology approach, metabolomics data and molecular docking simulation. The combination of TCM and modern analytical methods may provide new ideas for the study of TCM, and provide new therapeutic strategies and targets for liver fibrosis.

## Conclusions

The classical herb pair HQD is widely used in clinic for the treatment of liver fibrosis. In this study, we tried to understand the MoA of HQD on liver fibrosis for the purpose of utilizing it more safely and effectively. By combining metabolomics data, network pharmacology and molecular docking methods, we took prototype compounds and metabolites of HQD after administration together with their concentrations into consideration, and found that eight compounds (5-Hydroxysulfamethoxazole, Tauroursodeoxycholic acid, (S)-[8]-Gingerol, 4′,7,8-Trihydroxyisoflavanone, Calycosin, Genistein, Daidzein, and Liquiritin) and eight targets (CDK1, MMP9, PPARD, PPARG, PTGS2, SERPINE1, TP53, and HIF1A) might contribute to the effect of HQD on liver fibrosis by maintaining the balance of ECM, reducing liver damage, inhibiting hepatocyte apoptosis, and alleviating liver inflammation. This study provides a new way to investigate the MoA of Chinese medicine by considering the concentrations of components and metabolites.

## Supplementary Information

**Additional file 1. Table S1.** The information of 68 compounds.

**Additional file 2. Table S2.** Known and predicted component-target interactions.

**Additional file 3. Table S3.** The glide gscores of 29 PCTIs.

**Additional file 4. Table S4.** The glide gscores of cross docking.

## Data Availability

The data can be requested from the author upon reasonable request.

## Abbreviations

ALD: Alcoholic liver disease
AR: Androgen receptor
bSDTNBI: Balanced substructure-drug-target network-based inference
CDK1: Cyclin-dependent kinase 1
CLFT: Compound-liver fibrosis target
Clo: Closeness
CTI: Compound-target interaction
EC: EcCentricity
ECM: Extracellular matrix
EPC: Edge Percolated Component
Fc: Fold change
FC_MAX: Maximum of fold change
HBV: Hepatitis B virus
HCC: Hepatocellular carcinoma
HCV: Hepatitis C virus
HIF1A: Hypoxia-inducible factor-1α
HQD: Huangqi decoction
HSC: Hepatic stellate cell
IL1B: Interleukin-1 beta
IL6: Interleukin-6
MMP: Matrix metalloproteinase
MMP2: Matrix metalloproteinase-2
MMP9: Matrix metalloproteinase-9
MoA: Action of mechanism
NAFLD: Nonalcoholic fatty liver disease
NASH: Nonalcoholic steatohepatitis
NF-κB: Nuclear factor-kappa B
PPAR: Peroxisome proliferator-activated receptor
PPARA: Peroxisome proliferator-activated receptor alpha
PPARD: Peroxisome proliferator-activated receptor delta
PPARG: Peroxisome proliferator-activated receptor gamma
PPI: Protein–protein interaction
PTGS2: Prostaglandin G/H synthase 2
*R. Astragali*: *Radix Astragali*
Rad: Radiality
*R. Glycyrrhizae*: *Radix Glycyrrhizae*
SERPINE1: Plasminogen activator inhibitor-1
TCM: Traditional Chinese Medicine
TIMP: Tissue inhibitors of metalloproteinases
TNF: Tumor necrosis factor
TP53: Cellular tumor antigen p53
VDR: Vitamin D3 receptor
